# The impact of the herd health interventions in small ruminants in low input production systems in Ethiopia

**DOI:** 10.3389/fvets.2024.1371571

**Published:** 2024-10-21

**Authors:** Mesfin Mekonnen Moliso, Wassie Molla, Asrat Arke, Tesfalem Nana, Firdawok Ayele Zewudie, Abebe Tibebu, Aynalem Haile, Mourad Rekik, Ulf Magnusson, Barbara Wieland, Theodore Knight-Jones

**Affiliations:** ^1^Animal and Human Health Program, International Livestock Research Institute (ILRI), Addis Ababa, Ethiopia; ^2^College of Veterinary Medicine and Animal Sciences, University of Gondar, Gondar, Ethiopia; ^3^Bonga Agricultural Research Center, Bonga, Ethiopia; ^4^Areka Agricultural Research Centre, Areka, Ethiopia; ^5^Debre Berhan Agricultural Research Centre, Debre Berhan, Ethiopia; ^6^Sekota Dryland Agricultural Research Center, Sekota, Ethiopia; ^7^International Centre for Agricultural Research in the Dry Areas (ICARDA), Addis Ababa, Ethiopia; ^8^Department of Clinical Sciences, Swedish University of Agricultural Sciences, Uppsala, Sweden; ^9^Institute of Virology and Immunology, Mittelhäusern, Switzerland; ^10^Department of Infectious Diseases and Pathobiology, Vetsuisse Faculty, University of Bern, Bern, Switzerland

**Keywords:** herd health, small ruminants, respiratory diseases, community breeding, vaccination

## Abstract

**Introduction:**

Diseases have a negative impact on production and profitability of small ruminants. A good herd health program can decrease the number of sick animals and improve herd performance.

**Methods:**

In a longitudinal study, small ruminant herd health interventions such as community-based strategic gastrointestinal (GI) parasite control, prevention and control of major respiratory diseases and capacity development activities were implemented. In four districts of Ethiopia, where the Community Based Breeding Program (CBBP) is implemented, morbidity and mortality data were collected from January 2018 to July 2021 in 1047 smallholder farms with the objective of evaluating the impact of herd health interventions. A total of 2,643 sick animals and 516 deaths of small ruminants were recorded during the study period. The disease cases were categorized into eight groups: gastrointestinal, neurological, reproductive, respiratory, skin, systemic, other diseases (eye disease, foot disease etc) and unknown diseases. Chi-square and proportions were used to analyze morbidity and mortality by district, agro-ecological zone and age of the animal.

**Results:**

The data showed that the general trend in the occurrence of cases and morbidity rate were decreasing from 2018 to 2021 in intervention villages. Overall, the morbidity rate in young animals (7.36%) was highier than in adults (3.49%) and the mortality rate difference between young and adult animals was also statistically significant (*p* < 0.001). The morbidity and mortality rates varied significantly (*p* < 0.001) among districts and among agro-ecologies. According to the data, treating and following up of infected animals reduced the mortality rate significantly. The relative risk of death in treated animals after the case reported was 0.135. Generally, the intervention impact analysis revealed that morbidity rate was decreased in intervention years (6.31% in 2018 to 3.02% in 2021) and that herd health interventions provide an added value.

**Conclusion:**

Generally, herd health intervention had significant impact in reducing the morbidity rates in years and treatment and follow up of sick animals due to early reporting reduced mortality rate significantly. It is recommended that the herd health management should be the core activity under small ruminant production programs.

## Introduction

Sheep and goat production in Ethiopia is mainly carried out by smallholder farmers and pastoralists with an estimated population of 30.7 and 30.2 million sheep and goats, respectively (1).

According to previous studies, constraints to increased productivity of small ruminants in Ethiopia are primarily diseases and parasites, feed shortage, and inadequate extension service delivery or lack of improved breeding technologies (2). Diseases or parasite infestation was the most commonly identified constraint mentioned as a major challenge in over half (51.9%) of the studies performed in Ethiopia (3) and the annual mortality ranges from 12 to 14% for sheep and 11 to 13% for goats in Ethiopia (4). Recent reports showed up to 20% mortality of small ruminants between 2005 and 2015 (1, 5). The loss of animals, loss of productivity and value, treatment costs, migration of producers for other jobs, wastage of time to treat sick animals, malnutrition, the school dropout of children, human health, social and psychological impacts are the common impacts in Ethiopia of poor small ruminant health (6).

Le Blanc et al. (7) explained “A herd health program is an integrated, holistic, proactive, data-based, and economically framed approach to prevention of disease and enhancement of performance.” According to this definition the herd health programs consider all factors influencing livestock health including nutrition, environment and other management practices. Thus, a herd health program aims to significantly reduce morbidity and mortality. Individual animal health interventions may solve a specific disease problem but will not generate sufficient gains in animal health, welfare, production and productivity and any impact might be short-lived. In contrast, herd health programs have been demonstrated to improve livestock efficiency, address the productivity constraints and thereby the consumers gain from improved animal health (8, 9).

Morbidity and mortality data allows for continuous evaluation of the efficacy of either a specific health care system or an intervention implemented at herd and population level. Eventually, the observation of mortality together with morbidity allows the study of the burden a disease event may place on a population. These metrics also allow stakeholders to prioritize health problems and allocate resources toward and proactively manage the potential onset of health problems (10). Despite Ethiopia’s large livestock population, including small ruminants, and the high prevalence of animal diseases, the animal health service is steadily deteriorating, much like in many other African countries (11).

A participatory epidemiological study in sites where a Community Based Breeding Program (CBBP) was being implemented showed that respiratory diseases and gastrointestinal parasites are key constraints for animal health (6). The study also revealed the high negative impact of animal diseases on the livelihood of women, children and communities as a whole and there was no holistic approach in the sites to prevent and control the diseases. Once the health problems were identified, control measures to overcome the problems were formulated in stakeholder consultation and were combined into a community herd health program. This included respiratory disease interventions, gastrointestinal parasite and coenurosis control, reproductive performance improvement interventions and capacity development activities. The animal health interventions were integrated with other small ruminant packages such as breeding, feed and forage, grazing land (environment) management, marketing, gender and capacity development components. The interventions were carried out at the village level which use communal grazing land and intended to be undertaken with full engagement of farmers and other stakeholders even though the data was not collected from the control villages for the comparison due to various reasons. This intervention project aimed to decrease the morbidity and mortality of small ruminants and identify the major disease categories in the study areas. The aim of this study was to assess the impact of the intervention through key indicators such as morbidity and mortality rates.

## Materials and methods

### The study area

**Figure 1:**
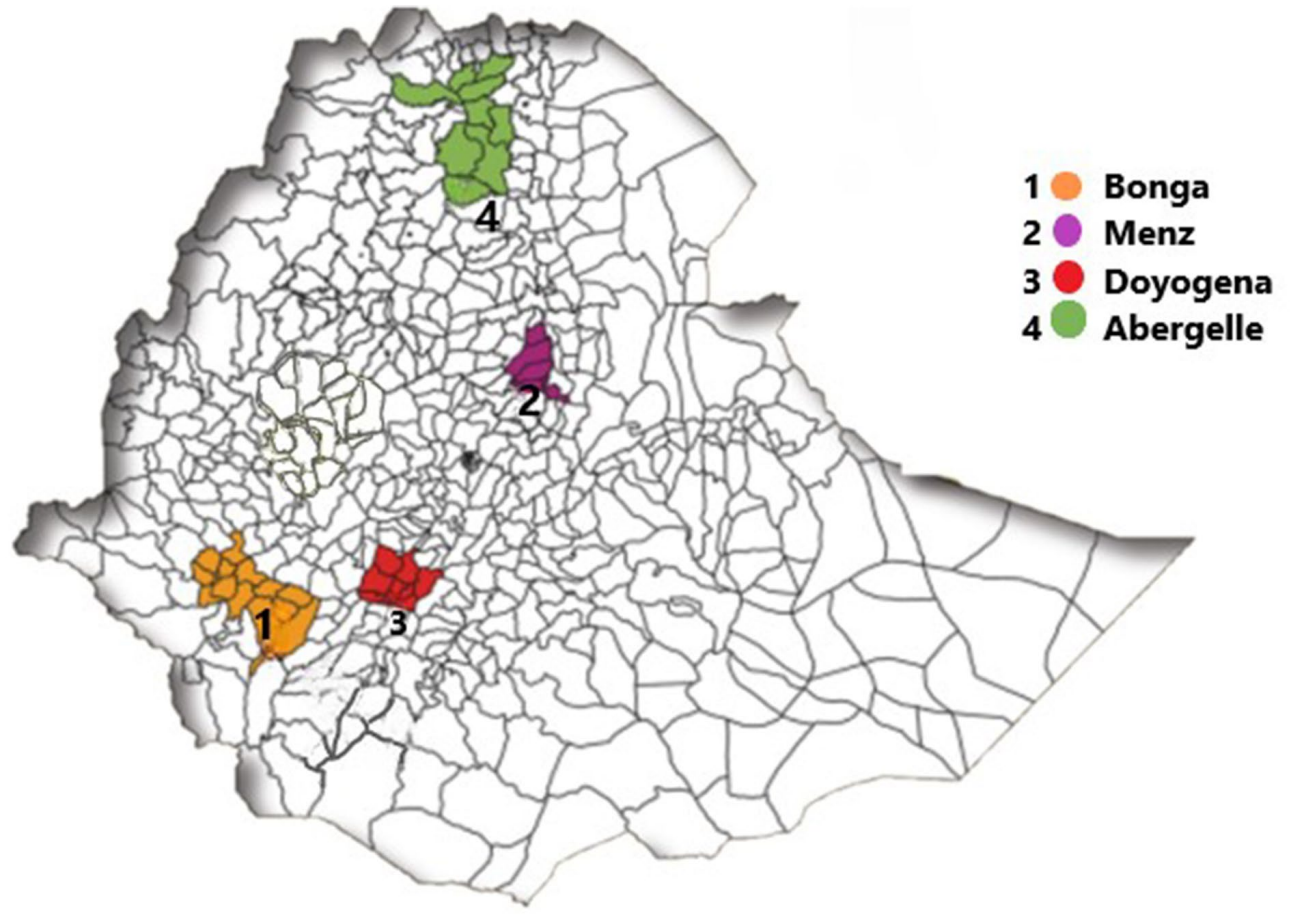
A map showing the community based small ruminants breeding sites of Ethiopia (adapted from the report by ICARDA).

The study was conducted in sites where the CGIAR Research Program (CRP) on Livestock had been implementing a Community Based Breeding Program (CBBP), which aimed at initiating systematic breeding at the community level, including an organized animal identification and recording of performance and pedigree data (12). In 2018, two villages (initial intervention villages) were selected from each Adiyo, Menz and Doyogena districts and one village was selected from Abergelle district. In 2020, one additional village (new intervention villages) was selected from Adiyo, Menz and Doyogena districts. The community-based sheep breeding program is implemented in the Adiyo, Menz, and Doyogena districts, while the goat breeding program is implemented in Abergelle district. Sedentary mixed crop-livestock system is practiced in all the study districts (Figure 1).

Adiyo district is located in Kaffa zone of the Southwestern Ethiopia region and situated within a longitude of 36° 47′ E and latitude of 7° 26’ N with an altitude ranging from 500 to 3,500 meters above sea level. The temperature in the area varies from 3°C to 36°C. Mixed crop-livestock production is the dominant farming system in the area (27). Boka, Shuta and Shena villages were included in this study.

Doyogena is the other study district in Central Ethiopia Region of Ethiopia located at 37°50′ E longitude and 7°20’ N latitude. The altitude of the district ranges from 1900 to 2,800 meters above sea level (28). Hawara Arara, Ancha Sadicho and Lemi Suticho villages were included in this study.

The third study district, Menz, in the North Shewa zone of the Amhara regional state is situated between 39^o^–44^o^ E longitude and 10^o^–24^0^ N latitude with an altitude of 3,354 meters above sea level. The district annually receives an average rainfall of 980 mm and its average temperature varies between 5°C to 18°C (29). The district is extremely highland in Ethiopia where crop production and the keeping larger animals are difficult due to the environment (13). Keyafer, Sinamba-Boda and Zeram villages were included in this study. The fourth study district, Abergelle is located in the Wag Hemra zone of the Amhara regional state and situated between 13°20′N and 38°58′E latitude and longitude, respectively. Its altitude ranges from 1,150 to 2,500 meters above sea level with an annual mean temperature of 23–43°C and rainfall of 250–750 mm, representing the semi-arid nature of the district (14) and one of the most draught affected areas in Ethiopia. Bilaque village was included for this study.

Different components of integrated herd health were implemented consecutively and/or in parallel depending on logistics. The components included: training of men and women farmers and development agents on monitoring and reporting of diseases and deaths, health monitoring of small ruminant flocks, investigating the impacts of preventive measures, and applying the existing vaccination and treatment options for prevention and control of major respiratory diseases in small ruminants. The animals were vaccinated in all study districts for major respiratory diseases such as ovine pasteurellosis, Peste des Petits Ruminants (PPR), contagious caprine pleuropneumonia (CCPP) and sheep and goat pox. In order to control parasite-related respiratory infections, deworming of small ruminants for lungworm was conducted. Furthermore, gastrointestinal parasite and coenurosis control interventions were carried out. A strategic anthelmintic treatment regime, which involved seasonal deworming, targeted treatment and accurate dosing based on body weight, was established for small ruminants. For the control of coenurosis in Menz and Adiyo districts, we conducted deworming of dogs with Praziquantel. Likewise, farmers, development agents, and district veterinarians were trained on reproductive health management of small ruminants. The vaccination and deworming were conducted on all small ruminants and dogs in the study villages as the same epidemiological unit, not just on the animals belonging to project participants.

For each site, an annual calendar (e.g., calendar) scheduling all activities was developed with the communities and the animal health service providers from the district agricultural or livestock and fish development offices, development agents, and regional agricultural research institutes. Longitudinal investigation of cases was conducted with the objectives of determining the magnitude of morbidity and mortality, categorizing the leading causes of morbidity and mortality, identifying the trend of morbidity and mortality and assessing the impact of community herd health interventions on small ruminant morbidity and mortality.

### Study design and study population

A longitudinal study was set up in the selected sites. In January 2018, seven villages (initial intervention villages: Keyafer and Sinamba-Boda villages from Menz district; Balique village from Abergelle district; Boka and Shuta villages from Adiyo district and Ancha Sadicho and Hawara Arara villages from Doyogena district) were enrolled into the intervention and later in 2020, three additional villages (New intervention villages: Zeram from Menz district, Shena from Adiyo district, and Lemi Suticho from Doyogena district) were enrolled into the intervention. All interventions covered all small ruminants in a village since they were considered as being part of the same epidemiological unit due to communal grazing. To monitor the impact of the interventions, a total of 100 households were closely followed in each village by the enumerators and animal health assistants recruited by the project to monitor the impact of the interventions. The enumerators, who visited the participant farmers at least once a week, were selected from the villages received and received training on the names, clinical signs, diagnosis, and reporting of the small ruminant diseases as well as animal health management. All small ruminants in the selected villages received animal health packages in addition to other interventions through the CRP Livestock program, such as feed and genetic improvement activities. The service costs for this intervention were covered by the project.

### Herd health interventions and data collection

In study villages, all small ruminants were systematically vaccinated against ovine pasteurellosis, PPR, Sheep and goat pox, Anthrax (in sites at risk) and Contagious Caprine Pleuropneumonia (CCPP). The vaccines were produced by National Veterinary Institute, NVI, Ethiopia. As all sites had high parasite burden, site strategic deworming campaigns with albendazole (Albentong 600, Congqing Fangtong Animal Pharmaceutical co. Ltd., China) for nematodes and Triclabendazole (Fascinex, Ciba-Geigy Ltd., Switzerland) for liver fluke were set up. These anthelminthics were administered together and given as per manufacturer recommendations. The vaccination, treatment and expert advice services were free and covered by the CRP livestock project.

To monitor impact, longitudinal household-level data on herd mortality and the incidence of respiratory, reproductive, gastro-intestinal tract and other diseases was recorded for 1,047 small ruminant herds during regular visits by trained village-based animal health enumerators. The participating farmers reported disease and death cases to the animal health enumerators, who directly consulted district level veterinarians and/or regional animal health researchers. The veterinarians diagnosed and treated the animal as soon (within hours) as they were reported. If the animal was, not treated it was mainly due to the report failure and it was recorded as having died without treatment. If the animal was cured without treatment due to reporting failure, it was still recorded. Data was collected on flock size, species, sex, age (young for below 6 months and adult for above 6 months), disease observation date, disease category, treatment status (treated or died before or without treatment), and date of death for animals died since the previous visit.

Diseases were categorized based on history of an animal and clinical signs observed or reported and local (vernacular) names into disease categories to minimize wrong specific diagnosis. Accordingly, the categories of diseases were respiratory, gastro-intestinal tract diseases, neurological, reproductive, skin diseases, systemic diseases, other minor cases and unknown cases (Table 1).

**Table 1:** Major disease categories and their clinical signs or local names.

S. no	Disease category	Major signs and/or local names of disease
1	Respiratory diseases	Respiratory signs such as sneezing, nasal discharge, dyspnoea, *‘engib’, ‘sal’, ‘oshiyo’, ‘kedefera’, ‘nift’*
2	Gastro-intestinal diseases	Diarrhea, emaciation, erected hair, ascites, bottle jaw, and presence of different stages of parasite in feces, *‘kizen’, ‘hod kurtet’, ‘kisat’, ‘kulkulit’, ‘mawule’, ‘zeso’*
3	Neurological diseases	Circling, abnormal gait, *‘azurit’*,
4	Reproductive disorders	Abortion, retained fetal membrane, dystocia, *‘yewolid chigr’, ‘wurja’*
5	Systemic diseases	Acute/sudden deaths*, ‘dingetegna’, ‘kenkento’*
6	Skin diseases	Itching, hair loss, crusts, irritation, wounds, *‘gogi-mosu’*
7	Other minor diseases	This category includes the following diseases and signs: eye diseases, foot rot, fracture of leg, ecto-parasites, mechanical injuries, draught or hunger - related cases, eaten by predator, dog bite, *‘mefzeze sewnet’, ‘sibrat’, ‘etie’*
8	Unknown syndromes	Clinical signs difficult to be classified, like stopping feeding, stretching body

### Data management and analysis

All data was entered, cleaned and stored in Microsoft Excel. Statistical analysis was performed using STATA-14 Software (Stata Corp, Texas, USA). Animal year at risk is the sum of the periods of observation for each animal during which the animal is free from the disease (i.e., is at risk) (15). Mortality rate refers to the frequency of death within a defined population over a specific time period, while morbidity rate refers to the frequency of occurrence of disease or health conditions within the same population and time frame. The population of small ruminants in the study villages for a year was determined by averaging the number of animals at the beginning and end of the year. Proportional morbidity is the proportion of animals affected by a specific disease or condition among all animals diagnosed with any disease during the study period, while proportional mortality denotes the proportion of animals that died from a particular disease or condition among all deaths from any disease during a specific time period. Proportion was employed to analyze the morbidity and mortality by district, village, agro-ecological zone, age category, disease category, and proportional morbidity and mortality. Descriptive statistics such as mean, and frequency were used to analyze the data. *Chi square* was employed to assess the differences in morbidity and mortality by age, and impact of treatment of sick animals on mortality. The impact of the implemented herd health program was evaluated by comparing the trend of mortality and morbidity in years. This was detected using the smoothed three-month moving average and regression analysis, as the data were time-series. The relative risk of death, which compares the probability of death occurring in one group to another, was calculated for treated animals and non-treated animals: the proportion of death in exposed group (treated group) over the proportion of death in un-exposed (non-treated).

## Results

### Morbidity and mortality rate in respect to district and village

Participating farms raised a total of 60′777 small ruminants (animal year at risk) in1047 herds and a total of 2,643 sick and 516 dead animals were recorded in the period January 2018 to July 2021. The data of 2,587 sick animals were complete whereas the data of the remaining 56 sick animals were incomplete either for sex, age, disease category or treatment. In general, a clear trend toward reduced morbidity was reported. The village level small ruminant morbidity rate in years is depicted in Appendix Table 3. The highest morbidity rate was reported in Ancha Sadicho in 2018 (33.5%), followed by Hawara Arara in the same year (29.38%). Mortality rate increased in some villages (Appendix Table 3), and the highest was recorded in in Lemi Suticho in 2020 (4%) and the second highest was recorded in Zeram (3.3%) in the same year.

Both morbidity and mortality were higher (*p* < 0.001) in young than adult animals. Village wise, the highest morbidity (24.3%) was documented in young sheep in Keyafer village, and the highest mortality rate (8.6%) was observed in young sheep in Lemi Suticho village in the study period (Appendix Table 1).

The morbidity and mortality rates were different (*p* < 0.001) among districts. The highest morbidity rate was reported in Doyogena district (31.5%) in 2018 while the highest mortality was documented in Menz district (1.9%) in 2020 (Table 2). The morbidity rate decreased from 6.31% in 2018 to 3.02% in 2021. There were also variations (*p* < 0.001) among agro-ecologies or districts. The highest morbidity as well as mortality were reported in wet mid highland areas (Table 2).

**Table 2:** Morbidity and mortality of small ruminants by districts in years, agro-ecological zones and species.

Districts	Species	Agro-ecology	Morbidity				Mortality				*p*-value (district)
			Years				Years				
			2018	2019	2020	2021	2018	2019	2020	2021	
Menz	Sheep	Sub moist HL	9.73	8.4	2.5	3.9	0.68	1.75	1.92	1.3	<0.001
Adiyo	Sheep	Wet MHL	1.2	0.15	2.45	1	0.2	0.15	1.07	0.04	
Doyogena	Sheep	Wet MHL	31.5	10	12.4	12.3	1.83	1.39	1.9	1.79	
Abergelle	Goat	Dry LL	7.6	9.3	11.45	6.32	0.74	0	0.17	0.08	
Total			6.31	5.4	3.57	3.02	0.5	0.87	1.38	0.54	

### Morbidity and mortality with respect to disease category and timeline

**Figure 2:**
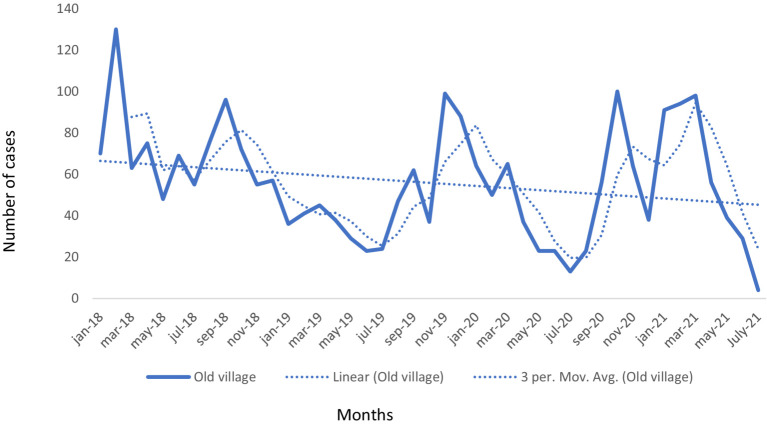
Occurrence and trends of small ruminant cases by month in initial villages (linear = trendline; 3 per. Mov. Avg. = 3 months moving average, Old village = initial intervention villages from 2018).

**Figure 3:**
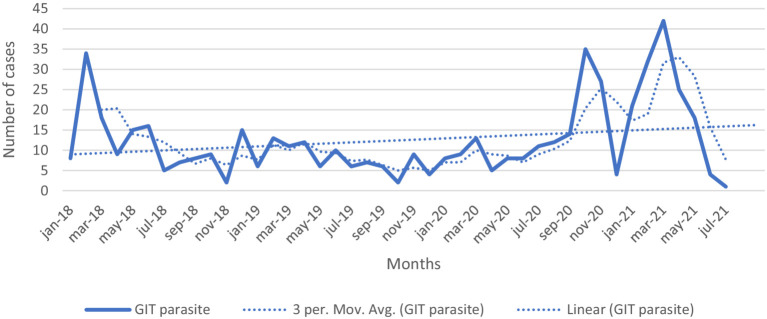
Occurrence and trends of gastrointestinal disease cases by month (linear = trendline; 3 per. Mov. Avg. = 3 months moving average).

The disease occurrence and changes over the study period are indicated in Figure 2. The general tendency in Figure 2 showed a decreasing trend in the occurrence throughout the intervention period. In general, based on the disease category relatively higher overall morbidity rate were due to respiratory (2.3%) and gastrointestinal tract (09%) diseases, with least cases attributed to the skin disease category (0.07%) in four study years (Table 3). We saw that the trend of occurrence of gastrointestinal related cases was increasing from year to year (Figure 3). At village level, the highest prevalence due to respiratory disease was reported in Ancha sadicho (31.75%) and Hawara Arara (21.46%) villages in 2018 whereas in gastrointestinal category the highest prevalence was observed in Hawara Arara (4.79%) and Keyafer (3.76%) villages in 2018 (Appendix Tables 2, 3). The highest number of deaths were attributed to respiratory and gastrointestinal diseases.

**Table 3:** Morbidity by disease category and village (Village with superscript ^G^ = goats).

Village	Total no. of animal year at risk	Disease category with number of cases and periodic prevalence (%)								Total cases
		Gastrointestinal	Neurological	Other	Reproductive	Respiratory	Skin disease	systemic	Unknown	
Ancha Sadicho	1,867	22 (1.18)	0 (0.00)	11 (0.59)	7 (0.37)	274 (14.68)	0 (0.00)	0 (0.00)	4 (0.21)	318 (17.03)
Bilaque^G^	4,890	13 (0.27)	1 (0.02)	55 (1.12)	57 (1.17)	211 (4.31)	31 (0.63)	45 (0.92)	7 (0.14)	420 (8.59)
Boka	17,210	140 (0.81)	37 (0.22)	24 (0.14)	6 (0.03)	85 (0.49)	0 (0.00)	23 (0.13)	24 (0.14)	339 (1.97)
Hawara Arara	1,841	38 (2.06)	0 (0.00)	12 (0.65)	26 (1.41)	244 (13.25)	0 (0.00)	0 (0.00)	2 (0.11)	322 (17.49)
Keyafer	7,532	192 (2.55)	47 (0.62)	31 (0.41)	10 (0.13)	339 (4.51)	8 (0.11)	25 (0.33)	13 (0.17)	665 (8.83)
Lemi Suticho	450	4 (0.89)	0 (0.00)	4 (0.89)	0 (0.00)	34 (7.56)	0 (0.00)	0 (0.00)	6 (1.33)	48 (10.67)
Shena	1,355	1 (0.07)	0 (0.00)	4 (0.30)	0 (0.00)	43 (3.17)	0 (0.00)	1 (0.07)	1 (0.07)	50 (3.69)
Shuta	12,601	12 (0.10)	5 (0.04)	4 (0.03)	4 (0.03)	12 (0.10)	0 (0.00)	4 (0.03)	0 (0.00)	41 (0.33)
Sinamba-Boda	8,564	50 (0.58)	8 (0.09)	2 (0.02)	1 (0.01)	92 (1.07)	0 (0.00)	7 (0.08)	79 (0.92)	239 (2.79)
Zeram	4,467	63 (1.41)	12 (0.27)	0 (0.00)	0 (0.00)	56 (1.25)	3 (0.07)	8 (0.180)	3 (0.07)	145 (3.25)
Overall	60,777	535 (0.88)	110 (0.18)	147 (0.24)	111 (0.18)	1,390 (2.29)	42 (0.07)	113 (0.19)	139 (0.23)	2,587 (4.26)

With respect to proportional morbidity, the highest was observed in the respiratory diseases (0.54) followed by the gastrointestinal diseases group (0.21). On the other hand, the highest proportional mortality rate was attributed to gastrointenstical causes (0.28) and respiratory causes (0.27) (Table 4).

**Table 4:** Proportional morbidity and mortality rates by disease category.

Disease category	No. of animals affected	Proportional morbidity rate	No. of animals died	Proportional mortality rate
Gastrointestinal	535	0.21	144	0.28
Neurological	110	0.04	61	0.12
Other	147	0.06	36	0.07
Reproductive	111	0.04	2	0.004
Respiratory	1,390	0.54	139	0.27
Skin disease	42	0.02	0	0
Systemic	113	0.04	19	0.04
Unknown	139	0.05	115	0.22
Total	2,587	1	516	1

### Impact of treatment on mortality

The morbidity and mortality data analysis showed that treating sick animals reduces mortality rate significantly (*p* < 0.001) (Table 5). The relative risk of death in treated animals was 0.135 (0.12/0.89). This means that treated animals were approximately 7.4 times (1/0.135) less likely to die compared to the non - treated animals (Table 5).

**Table 5:** Effect of treatment on mortality.

Treatment status	No. of sick animals	No. of animals died	Mortality	*p* value
Treated	1,208	141	0.12	*p* < 0.001
Non-treated	406	361	0.89	
Total	1,614	502	0.31	

## Discussion

In this study, the occurrence of disease cases in small ruminants in study sites was monitored over 4 years, and a decrease was observed from 2018 to 2021. Even though training on data recording was conducted, the data quality was not as desired and closer follow-up from the research team was hampered during the COVID-19 pandemic, due to movement restrictions, which resulted in 56 incomplete case recordings.

The overall mortality rate of small ruminants in four years was 0.85% (0.78–0.92%) and this is very low compared to the other national reports of 12–14% and 11–13% of mortality rate in sheep and goats, respectively (4) and 20% mortality of small ruminants (1, 5). This substantially low mortality rate suggests that the herd health interventions implemented during this period may have been highly effective. Other intervention studies elsewhere in the world also showed decreased morbidity and mortality rates of small ruminants following animal health interventions (16). And according to the results, treating infected animals reduces mortality rate significantly (*p* < 0.001). The relative risk of death in treated animals is 0.13 which means that treated animals were 1.15 times less likely to die as nontreated cases. This indicates that sick animals that were treated had a greater chance of recovery and survival than the non-treated cases. The reports from the community-based animal health workers (CAHWs) sheep and goat project in pastoralist districts of Ethiopia showed a sign of reduction in the impact of mange, helminthosis, contagious caprine pleuropneumonia, orf and non-specific diarrhea (17). Results from a study conducted by Ayatunde and his colleagues in Mali confirmed that feed and health interventions significantly improved small ruminant production (18).

The study revealed a huge difference in morbidity and mortality rates among districts. Notably, the morbidity rate in the Doyogena district was 31.5% in 2018, the highest recorded among the districts. This indicates that Doyogena was the most disease-prevalent district at the start of the herd health intervention activities. By the end of the study, the morbidity rate in this district had decreased to 12.3%, although this rate is still higher compared to other districts. This suggests that much attention should be given to the health of small ruminants in Doyogena district. Additionally, the movement of small ruminants to watering points and markets should be investigated in the future due to its significant impact on disease transmission (11) in this district. Both morbidity and mortality rates were lower in Adiyo district. This might be attributed to the overall management of the animals, such as nutrition, since the area receives sufficient rainfall compared to other districts.

In this study, mortality was higher in younger animals compared to adult animals and the difference between these two age categories was statistically significant (*p* < 0.001). This shows that high mortality rates affect young small ruminants in the study areas. For the success and sustainability of the community-based breeding programs, future research and interventions in the study areas should focus on the health management of young animals. Previous studies in Ethiopia and other parts of the world support the current finding, that the mortality is higher in young animals (19–22). However, in this study we did not assess the effectiveness of the herd health intervention per age groups. This omission limits the ability to understand whether the interventions are equally effective for all age categories or if certain age groups might require more targeted approaches.

The case occurrence of diseases and morbidity rate were decreasing from 2018 to 2021 even though this decrease in morbidity was not statistically supported. This reduction could be linked to the implementation of herd interventions during this period. Nevertheless, without robust statistical analysis, attributing the decrease solely to these interventions remains uncertain. The integrated herd health intervention had some impact in reducing the morbidity rate, but it did not decrease the mortality rate. The non-decline in mortality rate might have been due to a lack of reporting of some deaths around the beginning of the intervention activities or can be attributed to increased awareness of the society on the importance of promptly reporting their animals’ sickness to get them treated. Additionally, the study team believes that the COVID-19 pandemic might have impacted the strict follow-up of sick animals in addition to drought and other diseases for which no prevention was implemented.

Even though vaccination and deworming are the major animal health interventions in Ethiopia, their impact has rarely been evaluated and reported (23). In this study, the trend of occurrence for gastrointestinal disease problem showed a continued increase in some of the sites such as Sinamba Boda and Zeram villages of Menz district. Since no detailed disease investigation was conducted, the real cause of the disease is not clear. More in-depth follow-up of cases is thus advised for future interventions. Additionally, increase in reported gastrointestinal parasites, especially since October 2020, could reflect a change in attitude and increased awareness among farmers in the study villages or could reflect that the deworming strategy did not have the desired effect. Elsewhere, the review on the impact of prophylactic anthelmintic treatment in West Africa indicates that biannual anthelmintic treatment could reduce mortality of small ruminants from 8% in control to 3% in treated animals (24). The study conducted in the same area in the same period indicated that there was total elimination of animals with a high worm burden (EPG > 1,500), and elimination of a third of those with moderate infections, and there were also signs of emerging drug resistance (25).

Creating effective animal health systems is challenging in developing countries, including Ethiopia. The sustainability of herd health programs in small ruminant breeding villages is crucial. The animal health service costs in this intervention were covered by the CRP Livestock Project, which might have a negative impact when the project stops its support. However, the project formed village-level animal health multi-stakeholder platforms (MSPs) to ensure the continuity of herd health interventions (26). The effectiveness and activity of these platforms and the continuity of their work should be studied in the future.

We identified some constraints in this study. Firstly, there could be possible case reporting bias especially around the beginning of the study due to the awareness gap of the beneficiaries and readiness of enumerators to follow and collect the data. Secondly, we did not collect the data from the control villages. This did not happen because it was not possible to reach non-project sites due to different issues, such as recruitment of enumerators & also continuous follow-up and data collection without supporting the farmers was not possible. It is believed that without data from the control group, it is not possible to conclude that the observed effects were due solely to the intervention. We calculated the morbidity and mortality rates using the average annual population. A significant drawback of this approach is that these values can be dependent on population variations over time. This means that fluctuations in the population size, such as due to births or deaths, can affect the accuracy of the morbidity and mortality rates. These variations can lead to either overestimation or underestimation mortality and morbidity rates, which can complicate the interpretation of the results, particularly when assessing the impact of interventions. Additionally, there was case diagnosis bias since the morbidity and mortality data were fully recorded based on the history of animal and clinical signs not by the laboratory diagnosis which may have gaps in confirmation since some diseases share clinical signs.

## Conclusions and recommendations

Treatment and follow up of sick animals due to early reporting reduced mortality rate significantly. Generally, integrated animal health intervention had significant impact in reducing morbidity rates in years. Even though there was no reduction in mortality rate in this intervention, low mortality was recorded in intervention sites as compared to the previous national reports.

Herd health management should be the core activity under small ruminant production programs in community based breeding program villages and other small ruminant producing areas. The government and other development organizations should take the lead in the planning and implementation of the herd health approach to control and prevent major production health problems of small ruminants.

## Data Availability

The datasets presented in this study can be found in online repositories. The names of the repository/repositories and accession number(s) can be found at: https://data.ilri.org/portal/dataset/community-based-small-ruminants-health-management.
