# Erratum: The impact of the herd health interventions in small ruminants in low input production systems in Ethiopia

**DOI:** 10.3389/fvets.2024.1528018

**Published:** 2024-11-25

**Authors:** Frontiers Production Office

**Affiliations:** Frontiers Media SA, Lausanne, Switzerland

**Keywords:** herd health, small ruminants, respiratory diseases, community breeding, vaccination

Due to a production error, an outdated version of the accepted manuscript was typeset and subsequently published. The article has now been updated with the latest text.

The publisher apologizes for this mistake.

The original version of this article has been updated.

