# Supplementary material for: The impact of the herd health interventions in small ruminants in low input production systems in Ethiopia
Source: Front Vet Sci. 2024 Oct 21;11:1371571. doi: 10.3389/fvets.2024.1371571 (PMC11532125; doi:10.3389/fvets.2024.1371571)
Supplement: Supplementary file 1 [file Table_1.docx]

Table 1. Morbidity and mortality by village, species and age (Village with superscript ^G^ = goats).

| Village | Small ruminant population total in four years | | | | No. of cases | | Morbidity in % | | No. of deaths | | Mortality in % | |
| --- | --- | --- | --- | --- | --- | --- | --- | --- | --- | --- | --- | --- |
|  | **Species** | **Young** | **Adult** | **Total** | **Young** | **Adult** | **Young** | **Adult** | **Young** | **Adult** | **Young** | **Adult** |
| Keyafer | Ovine | 829 | 6703 | 7532 | 202 | 463 | 24.37 | 6.91 | 20 | 40 | 2.41 | 0.60 |
| Sinamba-Boda | Ovine | 1833 | 6731 | 8564 | 131 | 108 | 7.15 | 1.60 | 90 | 57 | 4.91 | 0.85 |
| Zeram | Ovine | 1079 | 3388 | 4467 | 56 | 89 | 5.19 | 2.63 | 46 | 53 | 4.26 | 1.56 |
| Bilaque^G^ | Caprine | 1296 | 3594 | 4890 | 146 | 274 | 11.27 | 7.62 | 7 | 5 | 0.54 | 0.14 |
| Boka | Ovine | 2895 | 14315 | 17210 | 136 | 202 | 4.70 | 1.41 | 65 | 52 | 2.25 | 0.36 |
| Shena | Ovine | 426 | 929 | 1355 | 21 | 29 | 4.93 | 3.12 | 0 | 0 | 0.00 | 0.00 |
| Shuta | Ovine | 1889 | 10712 | 12601 | 24 | 17 | 1.27 | 0.16 | 6 | 3 | 0.32 | 0.0003 |
| Ancha Sadicho | Ovine | 699 | 1168 | 1867 | 75 | 240 | 10.73 | 20.55 | 15 | 18 | 2.15 | 1.54 |
| Hawara Arara | Ovine | 736 | 1105 | 1841 | 61 | 257 | 8.29 | 23.26 | 7 | 18 | 0.95 | 1.63 |
| Lemi Suticho | Ovine | 93 | 357 | 450 | 15 | 31 | 16.13 | 8.68 | 8 | 6 | 8.60 | 1.68 |
|  | | **11775** | **49002** | **60'777** | **867** | **1710** | **7.36** | **3.49** | **264** | **252** | **2.24** | **0.51** |
| Overall | |  |  |  |  |  |  |  |  |  |  |  |
