# Supplementary material for: The impact of the herd health interventions in small ruminants in low input production systems in Ethiopia
Source: Front Vet Sci. 2024 Oct 21;11:1371571. doi: 10.3389/fvets.2024.1371571 (PMC11532125; doi:10.3389/fvets.2024.1371571)
Supplement: Supplementary file 2 [file Table_2.docx]

Table 2: The morbidity of small ruminants by years, disease categories and villages (Village with superscript ^G^ = goats).

| **Year** | **Village** | **No. of small ruminants monitored** | **Gastro-**  **intestinal** | **Neuro-logical** | **Other** | **Repro-ductive** | **Respiratory** | **Skin disease** | **Systemic** | **Unknown** | **Total cases** |
| --- | --- | --- | --- | --- | --- | --- | --- | --- | --- | --- | --- |
| **2018** | Ancha Sadicho | 504 | 8 (1.59) | 0 (0.00) | 1 (0.20) | 3 (0.60) | 160 (31.75) | 0 (0.00) | 0 (0.00) | 0 (0.00) | 172 (34.13) |
|  | Bilaque^G^ | 1210 | 1 (0.08) | 0 (0.00) | 4 (0.33) | 26 (2.15) | 53 (4.38) | 1 (0.08) | 7 (0.58) | 0 (0.00) | 92 (7.60) |
|  | Boka | 3974 | 29 (0.73) | 5 (0.13) | 5 (0.13) | 2 (0.05) | 22 (0.55) | 0 (0.00) | 3 (0.08) | 6 (0.15) | 72 (1.81) |
|  | Hawara Arara | 480 | 23 (4.79) | 0 (0.00) | 4 (0.83) | 11 (2.29) | 103 (21.46) | 0 (0.00) | 0 (0.00) | 0 (0.00) | 141 (29.38) |
|  | Keyafer | 1730 | 65 (3.76) | 25 (1.45) | 11 (0.64) | 7 (0.40) | 129 (7.46) | 3 (0.17) | 9 (0.52) | 1 (0.06) | 250 (14.45) |
|  | Shuta | 3742 | 6 (0.16) | 5 (0.13) | 2 (0.05) | 1 (0.03) | 6 (0.16) | 0 (0.00) | 2 (0.05) | 0 (0.00) | 22 (0.59) |
|  | Sinamba Boda | 2083 | 21 (1.01) | 2 (0.10) | 1 (0.05) | 0 (0.00) | 38 (1.82) | 0 (0.00) | 0 (0.00) | 18 (0.86) | 80 (3.84) |
| **2019** | Ancha Sadicho | 465 | 13 (2.80) | 0 (0.00) | 3 (0.65) | 0 (0.00) | 27 (5.81) | 0 (0.00) | 0 (0.00) | 1 (0.22) | 44 (9.46) |
|  | Bilaque^G^ | 1238 | 4 (0.32) | 0 (0.00) | 10 (0.81) | 28 (2.26) | 54 (4.36) | 7 (0.57) | 10 (0.81) | 2 (0.16) | 115 (9.29) |
|  | Boka | 4119 | 5 (0.12) | 1 (0.02) | 0 (0.00) | 0 (0.00) | 0 (0.00) | 0 (0.00) | 0 (0.00) | 0 (0.00) | 6 (0.15) |
|  | Hawara Arara | 540 | 6 (1.11) | 0 (0.00) | 3 (0.56) | 4 (0.74) | 47 (8.70) | 0 (0.00) | 0 (0.00) | 2 (0.37) | 62 (11.48) |
|  | Keyafer | 1852 | 57 (3.08) | 10 (0.54) | 5 (0.27) | 2 (0.11) | 171 (9.23) | 4 (0.22) | 7 (0.38) | 1 (0.05) | 257 (13.88) |
|  | Sinamba Boda | 2256 | 5 (0.22) | 2 (0.09) | 1 (0.04) | 1 (0.04) | 24 (1.06) | 0 (0.00) | 3 (0.13) | 41 (1.82) | 77 (3.41) |
| **2020** | Ancha Sadicho | 398 | 0 (0.00) | 0 (0.00) | 2 (0.50) | 3 (0.75) | 36 (9.05) | 0 (0.00) | 0 (0.00) | 2 (0.50) | 43 (10.80) |
|  | Bilaque^G^ | 1144 | 7 (0.61) | 1 (0.09) | 26 (2.27) | 3 (0.26) | 63 (5.51) | 11 (0.96) | 17 (1.49) | 3 (0.26) | 131 (11.45) |
|  | Boka | 4381 | 92 (2.10) | 30 (0.68) | 17 (0.39) | 4 (0.09) | 47 (1.07) | 0 (0.00) | 9 (0.21) | 15 (0.34) | 214 (4.88) |
|  | Hawara Arara | 400 | 4 (1.00) | 0 (0.00) | 1 (0.25) | 6 (1.50) | 65 (16.25) | 0 (0.00) | 0 (0.00) | 0 (0.00) | 76 (19.00) |
|  | Keyafer | 2136 | 18 (0.84) | 5 (0.23) | 4 (0.19) | 0 (0.00) | 21 (0.98) | 0 (0.00) | 2 (0.09) | 6 (0.28) | 56 (2.62) |
|  | Lemi Suticho | 200 | 2 (1.00) | 0 (0.00) | 1 (0.50) | 0 (0.00) | 3 (1.50) | 0 (0.00) | 0 (0.00) | 4 (2.00) | 10 (5.00) |
|  | Shena | 678 | 0 (0.00) | 0 (0.00) | 2 (0.29) | 0 (0.00) | 7 (1.03) | 0 (0.00) | 0 (0.00) | 0 (0.00) | 9 (1.33) |
|  | Shuta | 4240 | 0 (0.00) | 0 (0.00) | 0 (0.00) | 1 (0.02) | 4 (0.09) | 0 (0.00) | 0 (0.00) | 0 (0.00) | 5 (0.12) |
|  | Sinamba Boda | 2358 | 2 (0.08) | 4 (0.17) | 0 (0.00) | 0 (0.00) | 9 (0.38) | 0 (0.00) | 1 (0.04) | 14 (0.59) | 30 (1.27) |
|  | Zeram | 2520 | 32 (1.27) | 11 (0.44) | 0 (0.00) | 0 (0.00) | 37 (1.47) | 0 (0.00) | 5 (0.20) | 2 (0.08) | 87 (3.45) |
| **2021** | Ancha Sadicho | 500 | 1 (0.20) | 0 (0.00) | 5 (1.00) | 1 (0.20) | 51 (10.20) | 0 (0.00) | 0 (0.00) | 1 (0.20) | 59 (11.80) |
|  | Bilaque^G^ | 1298 | 1 (0.08) | 0 (0.00) | 15 (1.16) | 0 (0.00) | 41 (3.16) | 12 (0.92) | 11 (0.85) | 2 (0.15) | 82 (6.32) |
|  | Boka | 4736 | 14 (0.30) | 1 (0.02) | 2 (0.04) | 0 (0.00) | 16 (0.34) | 0 (0.00) | 11 (0.23) | 3 (0.06) | 47 (0.99) |
|  | Hawara Arara | 421 | 5 (1.19) | 0 (0.00) | 4 (0.95) | 5 (1.19) | 29 (6.89) | 0 (0.00) | 0 (0.00) | 0 (0.00) | 43 (10.21) |
|  | Keyafer | 1814 | 52 (2.87) | 7 (0.39) | 11 (0.61) | 1 (0.06) | 18 (0.99) | 1 (0.06) | 7 (0.39) | 5 (0.28) | 102 (5.62) |
|  | Lemi Suticho | 250 | 2 (0.80) | 0 (0.00) | 3 (1.20) | 0 (0.00) | 31 (12.40) | 0 (0.00) | 0 (0.00) | 2 (0.80) | 38 (15.20) |
|  | Shena | 677 | 1 (0.15) | 0 (0.00) | 2 (0.30) | 0 (0.00) | 36 (5.32) | 0 (0.00) | 1 (0.15) | 1 (0.15) | 41 (6.06) |
|  | Shuta | 4619 | 6 (0.13) | 0 (0.00) | 2 (0.04) | 2 (0.04) | 2 (0.04) | 0 (0.00) | 2 (0.04) | 0 (0.00) | 14 (0.30) |
|  | Sinamba Boda | 1867 | 22 (1.18) | 0 (0.00) | 0 (0.00) | 0 (0.00) | 21 (1.12) | 0 (0.00) | 3 (0.16) | 6 (0.32) | 52 (2.79) |
|  | Zeram | 1947 | 31 (1.59) | 1 (0.05) | 0 (0.00) | 0 (0.00) | 19 (0.98) | 3 (0.15) | 3 (0.15) | 1 (0.05) | 58 (2.98) |
| **Total** | | **60’777** | **535**  **(0.88)** | **110**  **(0.18)** | **147**  **(0.25)** | **111**  **(0.18)** | **1’390**  **(2.29)** | **42**  **(0.07)** | **113**  **(0.19)** | **139**  **(0.23)** | **2’587**  **(4.26)** |
