# Supplementary material for: The impact of the herd health interventions in small ruminants in low input production systems in Ethiopia
Source: Front Vet Sci. 2024 Oct 21;11:1371571. doi: 10.3389/fvets.2024.1371571 (PMC11532125; doi:10.3389/fvets.2024.1371571)
Supplement: Supplementary file 3 [file Table_3.docx]

Table 3. Morbidity and mortality of small ruminant at village level (Village with superscript ^G^ = goats).

| Region | District | Village | Year | No. of small ruminants monitored | No of sick animals | Morbidity in % | No. of dead animals | Mortality in % |
| --- | --- | --- | --- | --- | --- | --- | --- | --- |
| Amhara | Menz | Keyafer | 2018 | 1730 | 255 | 14.74 (13.07-16.41) | 0 | 0.00 (0.00-0.00) |
|  |  |  | 2019 | 1852 | 259 | 13.98 (12.41-15.56) | 6 | 0.32 (0.07-0.58) |
|  |  |  | 2020 | 2136 | 56 | 2.62 (1.94-3.30) | 17 | 0.80 (0.42-1.17) |
|  |  |  | 2021 | 1814 | 104 | 5.73 (4.66-6.80) | 37 | 2.04 (1.40-2.69) |
|  |  | Sinamba-Boda | 2018 | 2083 | 116 | 5.57 (4.58-6.55) | 26 | 1.25 (0.77-1.73) |
|  |  |  | 2019 | 2256 | 88 | 3.90 (3.10-4.70) | 66 | 2.93 (2.23-3.62) |
|  |  |  | 2020 | 2358 | 34 | 1.44 (0.96-1.92) | 34 | 1.44 (0.96-1.92) |
|  |  |  | 2021 | 1867 | 58 | 3.12 (2.32-3.89) | 21 | 1.12 (0.65-1.60) |
|  |  | Zeram | 2020 | 2520 | 87 | 3.45 (2.74-4.17) | 84 | 3.33 (2.63-4.03) |
|  |  |  | 2021 | 1947 | 58 | 2.98 (2.22-3.73) | 15 | 0.77 (0.38-1.16) |
|  | Ziquala | Bilaque^G^ | 2018 | 1210 | 92 | 7.60 (6.11-9.10) | 9 | 0.74 (0.26-1.23) |
|  |  |  | 2019 | 1238 | 115 | 9.29 (7.67-10.91) | 0 | 0.00 (0.00-0.00) |
|  |  |  | 2020 | 1144 | 131 | 11.45 (9.61-13.30) | 2 | 0.17 (-0.07-0.42) |
|  |  |  | 2021 | 1298 | 82 | 6.32 (4.99-7.64) | 1 | 0.08 (-0.07-0.23) |
| SNNPR | Adiyo | Boka | 2018 | 3974 | 71 | 1.79 (1.37-2.20) | 7 | 0.18 (0.05-0.31) |
|  |  |  | 2019 | 4119 | 6 | 0.15 (0.03-0.26) | 6 | 0.15 (0.03-0.26) |
|  |  |  | 2020 | 4381 | 214 | 4.88 (4.25-5.52) | 100 | 2.28 (1.84-2.72) |
|  |  |  | 2021 | 4736 | 47 | 0.99 (0.71-1.27) | 4 | 0.08 (0.00-0.17) |
|  |  | Shena | 2020 | 678 | 9 | 1.33 (0.47-2.19) | 0 | 0.00 (0.00-0.00) |
|  |  |  | 2021 | 677 | 41 | 6.06 (4.26-7.85) | 0 | 0.00 (0.00-0.00) |
|  |  | Shuta | 2018 | 3742 | 22 | 0.59 (0.34-0.83) | 9 | 0.24 (0.08-0.40) |
|  |  |  | 2020 | 4240 | 5 | 0.12 (0.01-0.22) | 0 | 0.00 (0.00-0.00) |
|  |  |  | 2021 | 4619 | 14 | 0.29 (0.14-0.45) | 0 | 0.00 (0.00-0.00) |
|  | Doyogena | Ancha Sadicho | 2018 | 504 | 169 | 33.53 (29.41-37.65) | 9 | 1.79 (0.63-2.94) |
|  |  |  | 2019 | 465 | 42 | 9.03 (6.43-11.64) | 6 | 1.29 (0.26-2.32) |
|  |  |  | 2020 | 398 | 44 | 11.06 (7.97-14.14) | 9 | 2.26 (0.80-3.72) |
|  |  |  | 2021 | 500 | 60 | 12.00 (9.15-14.85) | 9 | 1.80 (0.63-2.97) |
|  |  | Hawara Arara | 2018 | 480 | 141 | 29.38 (25.30-33.45) | 9 | 1.88 (0.66-3.09) |
|  |  |  | 2019 | 540 | 59 | 10.93 (8.29-13.56) | 8 | 1.48 (0.46-2.50) |
|  |  |  | 2020 | 400 | 72 | 18.00 (14.24-21.77) | 2 | 0.50 (0.00-1.19) |
|  |  |  | 2021 | 421 | 46 | 10.93 (7.95-13.91) | 6 | 1.43 (0.29-2.56) |
|  |  | Lemi Suticho | 2020 | 200 | 8 | 4.00 (1.28-6.72) | 8 | 4.00 (1.28-6.72) |
|  |  |  | 2021 | 250 | 38 | 15.20 (10.75-19.65) | 6 | 2.40 (0.50-4.30) |
